# Use of Virtual Reality (VR)-Based Relaxation Among Female Patients with Mental Disorders: A Pilot Study

**DOI:** 10.3390/reports8040247

**Published:** 2025-11-26

**Authors:** Magdalena Stencel, Błażej Pilarski, Julia Kuca, Natalia Kapuśniak, Dorota Turska-Czyż, Szymon Florek, Magdalena Piegza, Piotr Gorczyca, Robert Pudlo

**Affiliations:** 1 Doctoral School, Department of Psychoprophylaxis, Faculty of Medical Sciences in Zabrze, Medical University of Silesia in Katowice, 42-612 Tarnowskie Góry, Poland; d201328@365.sum.edu.pl (M.S.); d201331@365.sum.edu.pl (B.P.); 2 Student’s Research Group, Department of Psychiatry, Faculty of Medical Sciences in Zabrze, Medical University of Silesia in Katowice, 42-612 Tarnowskie Góry, Poland; s82926@365.sum.edu.pl; 3 Psychiatric Department, Multidisciplinary Hospital in Tarnowskie Góry, 42-612 Tarnowskie Góry, Poland; n.e.kapusniak@gmail.com; 4 Department of Psychoprophylaxis, Faculty of Medical Sciences in Zabrze, Medical University of Silesia in Katowice, 42-612 Tarnowskie Góry, Poland; dturska@sum.edu.pl (D.T.-C.);; 5 Department of Psychiatry, Faculty of Medical Sciences in Zabrze, Medical University of Silesia in Katowice, 42-612 Tarnowskie Góry, Poland; mpiegza@sum.edu.pl (M.P.);

**Keywords:** virtual reality, Schultz autogenic training, relaxation, anxiety, depression, insomnia, cognitive functions, pilot study

## Abstract

**Background**: Anxiety disorders, depressive disorders, and insomnia often co-occur and impair functioning in psychiatric patients. Virtual reality (VR) is a promising relaxation tool, yet its efficacy relative to classical Schultz autogenic training (AT) remains insufficiently characterized. **Methods**: Thirty-seven female patients were randomly assigned to four groups: (1) inpatient AT (*n* = 10), (2) inpatient VR (*n* = 10), (3) home-based AT (*n* = 10), and (4) home-based VR (*n* = 7). Interventions lasted 2 weeks (≥10 sessions). Depressive and anxiety symptoms, sleep quality, and cognitive function were assessed pre- and post-intervention. **Results**: In the total sample, anxiety and depressive symptoms decreased and sleep quality improved, while cognitive functions showed slight improvement. In subgroup analyses, inpatient AT reduced anxiety and improved sleep, whereas inpatient VR reduced both anxiety and depressive symptoms and improved sleep. In the home-based modality, AT did not significantly affect sleep, anxiety, or depressive symptoms but was associated with modest cognitive gains, while home-based VR improved sleep without significant changes in anxiety or depression. **Conclusions**: Both methods are straightforward to implement and promote improvement in selected mental health parameters; however, their effect profiles are context-dependent. Interventions delivered in the inpatient setting were more effective than those at home, suggesting a substantial influence of contextual factors (fewer distractions, therapeutic structure, group component). Among the tested conditions, inpatient VR-based relaxation produced the broadest pattern of improvement in anxiety, depression, and sleep. These pilot findings require confirmation in larger, prospectively designed studies.

## 1. Introduction

Individuals with mental disorders experience a substantial deterioration in quality of life, decreased occupational productivity, social isolation, and an increased risk of premature death, both from somatic causes and by suicide [1]. According to data from the World Health Organization (WHO), since the onset of the COVID-19 pandemic, there has been a global deterioration in mental health. Findings from the June 2023 Eurobarometer survey indicate that, in the preceding twelve months, nearly half of European Union residents (46%) experienced emotional or psychosocial difficulties, including, among others, depressive symptoms and anxiety symptoms [2]. Cognitive dysfunction is part of the symptomatology of depressive disorders and may persist to varying degrees even during symptomatic remission. Persistent depressive symptoms in the form of cognitive dysfunction are associated with reduced quality of life, lower responsiveness to pharmacotherapy, and increased expenditures from public funds [3]. Insomnia is also a significant clinical problem, frequently co-occurring with mood disorders, anxiety disorders, and other psychiatric conditions. Its prevalence in the general population ranges from 6% to 30%, and it more commonly affects women and older adults. In Poland, this problem is estimated to affect up to half of adults, whereas in Europe the lowest rate has been reported in Germany (5.7%) and the highest in France (19%) [4]. 

It has become necessary to intensify efforts aimed at developing effective therapeutic methods, including non-pharmacological ones. Currently recognized non-pharmacological treatments for anxiety–depressive disorders include, among others, cognitive–behavioral therapy [5,6], biological interventions such as transcranial magnetic stimulation (TMS), naturopathic interventions encompassing phytotherapy, dietary supplementation, transcranial direct current stimulation (tDCS), and acupuncture, as well as light therapy [7,8]. The latest findings indicate a significant role of the gut microbiota in maintaining mental health. The microbiota–gut–brain axis is a complex, bidirectional communication system between the nervous system and the gastrointestinal tract, revealing close interdependencies between these domains. A detailed understanding of the composition of the microbiota and the mechanisms regulating these connections may, in the future, enable the development of innovative therapies that combine mental health care with the treatment of gastrointestinal disorders [9]. Another non-pharmacological therapeutic tool shown to be effective in alleviating depressive symptoms, reducing anxiety, and improving sleep quality is relaxation training [10,11]. This technique is based on mechanisms of self-regulation of the autonomic nervous system and employs passive concentration of attention. Despite the broad range of available therapeutic methods, the need to seek new, innovative approaches remains important. Of particular importance in this context is the active harnessing of the potential offered by modern technologies. Integrating technological solutions into therapeutic practice may enhance treatment effectiveness, allow better tailoring of interventions to individual patient needs, and expand the feasibility of delivering therapy across diverse settings, including outside the traditional clinical environment. 

As a credible comparator for VR-based relaxation, autogenic training according to Schultz was employed as the control condition. As a widely recognized and empirically validated method, Schultz autogenic training provided a robust reference point for determining whether the novel technique performs at least as effectively as a traditional approach [12]. An additional rationale was the versatility of AT: its capacity for standardized delivery in both clinical settings and home environments ensured methodological consistency across all phases of the study [13,14].

The application of modern technologies in psychiatry is at an early stage of development, and only a few centers are implementing innovative technological solutions. VR headsets (virtual reality) enable full immersion in a virtual environment, providing a high level of immersion that facilitates effective relaxation. Isolation from external stimuli may help reduce symptoms of depression, anxiety, and stress, improving quality of life and potentially lowering the risk of suicide [15]. VR headsets allow relaxation therapy to be tailored to the individual needs of the user by simulating natural environments, including outside the therapeutic center, which makes it possible to conduct relaxation sessions at home. The use of this form of therapy contributes to mood improvement and a reduction in anxiety levels [16]. Moreover, virtual reality technology makes it possible to construct interactive, three-dimensional, computer-generated simulations of social situations that remain under the operator’s full control. The use of VR headsets enables more immersive and engaging therapeutic interventions based on breathing training, whose efficacy in reducing depressive and anxiety symptoms has been confirmed in numerous studies, including among patients after hematopoietic stem cell transplantation and in pulmonary, cardiac, and neurological rehabilitation, particularly in patients with coronary artery disease and those after stroke [17,18,19]. Immersion refers to the objective system properties of VR that provide a multisensory, absorbing environment while limiting external stimuli. Presence is the subjective experience of “being in” the virtual space, arising from deep perceptual and emotional engagement [20]. Together, these mechanisms enhance affect regulation, support sleep preparation, and facilitate the acquisition of self-regulatory skills more effectively than audio-guided Schultz autogenic training alone [21]. Evidence supporting immersion as an active mechanism comes from research demonstrating that immersive VR can enhance engagement and cognitive processing; for example, Kubr et al.showed that specific immersive elements such as audio guidance and visual management cues improved short-term memory retention in an industrial VR training context. Although the application differs from clinical relaxation, the underlying mechanisms of heightened attentional focus and strengthened presence provide a plausible basis for similar benefits in therapeutic VR interventions [22].

Studies suggest a beneficial effect of VR therapy in depressive and anxiety disorders, pain perception, specific phobias, and post-traumatic stress disorder [23,24,25]. An important research question appears to be the analysis of the potential benefits of adjunctive use of virtual reality-based therapy in the context of limiting pharmacotherapy, thereby reducing the risk of adverse effects, while simultaneously assessing possible side effects directly associated with the use of VR technology [24]. 

The primary aim of the study is to conduct a comparative assessment of the effectiveness of Autogenic Training and virtual-reality-based relaxation in reducing psychopathological symptoms, including depressive and anxiety symptoms, in female psychiatric patients. The study further evaluates the impact of both interventions on subjective and objective sleep quality, examines their effects on selected cognitive functions such as attention, working memory, and executive functioning, and assesses differences in intervention efficacy across therapeutic contexts, specifically inpatient versus home-based settings. The study will examine the hypothesis that VR-based relaxation may lead to greater improvements in depressive symptoms, anxiety, sleep quality, and self-regulatory or cognitive outcomes compared with autogenic training. It will also investigate the possibility that these effects may be stronger in the inpatient setting than in home delivery, owing to more controlled and less variable therapeutic conditions. 

## 2. Materials and Methods

Female patients hospitalized in the Clinical Department of Psychiatry and outpatients treated at home for mental disorders were eligible for participation. Inclusion criteria were age ≥ 18 years and meeting the diagnostic criteria for at least one of the following ICD-11 categories: mood disorders (6A60–6A8Z), anxiety and fear-related disorders (6B00–6B0Z), obsessive–compulsive disorders (6B20–6B2Z), disorders specifically associated with stress (6B40–6B4Z). For hospitalized patients, psychiatric diagnoses were cross-checked against data in the patient’s discharge summary. For outpatients, the diagnosis established by the treating psychiatrist was considered binding. A member of the research team contacted potential participants, provided detailed information about the study, verified eligibility criteria, and obtained informed consent to participate. 

Exclusion criteria included: failure to comply with equipment use instructions, absence from more than six sessions, and contraindications to VR such as epilepsy, a history of cataract surgery, and the presence of electronic implants. 

### 2.1. Study Groups

Hospitalized patients were randomly assigned to one of two groups—the group performing standard relaxation using Schultz autogenic training and the group performing relaxation with VR headsets. The study used simple randomization: female participants were randomly allocated to groups upon presentation for enrollment. Each group comprised two to five participants at a time. Ten relaxation sessions were conducted over two weeks.

Similarly, outpatients were divided into two groups—the first performing Schultz autogenic training independently at home and the second performing VR-based relaxation independently at home. Participants in both outpatient groups were instructed to complete a minimum of 10 relaxation sessions over two weeks.

During the study, all patients continued their existing pharmacotherapy. Abrupt changes to patient management were avoided whenever clinically feasible, and all female participants received routine inpatient psychological care as part of standard ward practice. At home, treatment regimens were kept stable, and no formal psychological support was provided. Both the Schultz method and the use of VR headsets constituted adjunctive interventions. Pharmacotherapy was not modified during the study period. 

Only female patients participated in this pilot study. This decision had methodological justification: restricting the sample to one sex yielded a more homogeneous group and limited variability related to potential sex differences in clinical presentation, stress responses, and preferences and acceptance of VR technology. Moreover, the pilot aimed for a preliminary assessment of the feasibility, tolerability, and subjective utility of VR relaxation techniques in a female population in which certain mental disorders are reported more frequently, facilitating evaluation of the intervention’s usefulness. However, this restriction to women also limits the generalizability of the findings. In subsequent study phases, recruitment is planned to be extended to male wards to assess the generalizability of the findings. 

### 2.2. Interventions

Prior to study initiation, all investigators underwent standardized training delivered by a clinical psychologist and a psychiatrist, covering the delivery of each intervention and the identification and management of potential adverse events.

#### 2.2.1. Schultz Method in the Inpatient Setting

The control intervention consisted of relaxation training using Schultz autogenic training. An audio recording prepared by a psychologist contained a full relaxation session lasting 16 min. 

The relaxation session comprised the following components: introduction, breath-focused orientation, heaviness formulas, warmth formulas, autonomic (cardiorespiratory) regulation, and closure. No breathing cadence was prescribed; participants breathed at a self-selected pace. The complete script, in both Polish and English, is provided in the Supplementary Materials. 

Relaxation was conducted in a dedicated relaxation room with standardized conditions: indirect lighting, ambient noise < 40 dB (A), temperature 21–23 °C. 

Before each session, the facilitator provided brief instructions on posture, safety, and “passive concentration,” initiated the recording, and remained in the room throughout. 

During group inpatient sessions, patients lay on mats and listened to the recording.

#### 2.2.2. Schultz Method in the Home Setting

Participants performing the training at home received the identical recording on a mobile device and were instructed to play the session daily for two weeks. 

Additionally, participants received instructions on the conditions for conducting relaxation sessions (a room with ambient lighting, a quiet setting, and the absence of distractions). 

#### 2.2.3. VR-Based Relaxation in the Inpatient Setting

The experimental group used TierOne VR headsets (Świebodzka 41, 58-141 Stanowice, Poland). The equipment contained a set of immersive spherical films with a relaxation voiceover in Polish and an audio track (relaxation music or nature sounds). Sessions were conducted with participants seated and without forced locomotion. The relaxation content consisted of 360° videos, during which participants were instructed to calmly scan the surrounding scene at a comfortable pace. The list of available materials included the following collection of films: State of Balance, The art of Mastery, Mountain valley, Forest hike, Seaside Beach, Snowy lane, Therapeutic rebirth, Rocky Coast, Underwater World, Serenity of the Lake, Dawn Meditation, Twilight Meditation, Breathing Training, Walk in the Clouds, Natural Soothing. Each film lasted 10–15 min. 

Inpatient sessions were delivered in a dedicated relaxation room (the same as in the VR intervention). Participants were seated on stable chairs with backrests, spaced ≥ 1.5 m, with trip hazards removed. 

Sessions were supervised by trained staff present at all times. An immediate stop protocol and participant-initiated discontinuation were available (for dizziness, nausea, visual discomfort, psychological distress). Prior to the first session, participants were briefed on potential cybersickness symptoms and reporting procedures. 

Before each session we performed a standardized fit procedure: (1) strap and facial interface adjustment, (2) sharpness/comfort check, (3) volume setting. 

After every session, non-optical surfaces were disinfected with ~70% isopropyl alcohol wipes; lenses were cleaned only with alcohol-free optical wipes. Disposable sanitary covers were used for the facial interface. 

#### 2.2.4. VR-Based Relaxation in the Home Setting

Patients participating in the home study received the headset for a two-week period. Participants were instructed to select a quiet room with indirect lighting, a chair with back support, a 2 × 2 m clear area, and recommended presence of a household member during the first session. Patients were also thoroughly instructed regarding use of VR headsets and the session schedule (one per day). 

#### 2.2.5. Attendance Reporting

Attendance at sessions was systematically documented. Home-based participants were instructed to maintain a session log. During follow-up, they reported to the assessor—via a structured interview—the number of sessions completed and any relevant contextual details. 

### 2.3. Research Instruments

The effectiveness of the interventions was assessed using the following psychometric tools, administered before and after the intervention:

- Hospital Anxiety and Depression Scale (HADS)
- Athens Insomnia Scale (AIS)
- Addenbrooke’s Cognitive Examination III (ACE-III)
- Montreal Cognitive Assessment (MoCA)

We used the validated Polish version of the HADS. The 14-item scale yields anxiety and depression subscales. Items were rated on a four-point Likert scale (0–3). Each subscale produces a total score from 0 to 21. Polish studies show good internal consistency and criterion validity [26,27,28]. The Cronbach’s alpha coefficient in our study was 0.91, while the stability estimated using the test-retest method was for anxiety subscale 0.75 and depression subscale 0.71. 

Sleep was assessed using the 8-item Athens Insomnia Scale (AIS). Each item is rated on a four-point Likert-type scale (0 = absence of the symptom; 3 = marked severity). The original validation demonstrated high internal consistency (α ≈ 0.90) and very good test-retest reliability [29,30]. The Cronbach’s alpha coefficient in our study for the AIS was 0.83 and test-r, and the test-retest method gave a result test-retest method give a result of 0.50. 

Cognitive function was assessed using two instruments: the Montreal Cognitive Assessment (MoCA) and the Addenbrooke’s Cognitive Examination–III (ACE-III). The MoCA is a brief cognitive screening tool that provides a global index of cognitive status. It comprises tasks assessing visuospatial/executive abilities, naming, memory, attention, language, abstraction, delayed recall, and orientation. Total scores range from 0 to 30, with higher scores indicating better cognitive performance. In this study, the Polish-language version was used; prior studies support its diagnostic utility in Polish populations [31,32]. The stability of the tool in our study, estimated using the test-retest method, was 0.85. 

The ACE-III is a cognitive screening instrument. Scores range from 0 to 100 and are allocated across five domains: attention/orientation (18 points), memory (26), verbal fluency (14), language (26), and visuospatial abilities (16). Validation studies support its validity and demonstrate high sensitivity and specificity. The Polish-language version was used in the present study [33,34,35]. For the ACE-III total score, the test stability in our study, estimated using the test-retest method, was 0.82. 

### 2.4. Data Processing and Analysis

We performed quality checks (range, duplicates, timestamp consistency). Questionnaire scores (HADS, AIS, MoCA, ACE-III) followed official scoring rules. 

Statistical analysis was performed using Excel 365 and Statistica 13.3. The Shapiro-Wilk W test was used to assess distribution normality. The alpha-Cronbach test and the test-retest method using Pearson’s correlation were used to assess the stability of the tools used. Across the entire study and in all subgroups, variables related to cognitive functions (ACE-III, MoCA) were not normally distributed. The remaining variables followed a normal distribution. Comparative analyses of dependent variables were conducted: for normally distributed variables, Student’s *t*-test was applied, whereas for non-normally distributed variables, the sign test was used. Significance threshold α = 0.05 was adopted. 

## 3. Results

### 3.1. Characteristics of the Study Sample

Thirty-seven female patients participated in the study between 1 July 2024 and 30 June 2025.

The mean age of the participants was 41 years (standard deviation 18.623). The groups of patients undergoing standard Schultz-based relaxation in the inpatient setting and at home, as well as the group undergoing inpatient VR-based therapy, each comprises 10 participants. In contrast, the home-based VR group comprises 7 participants. The largest number of patients had a diagnosis of depressive disorder. Table 1 presents a detailed characterization of sociodemographic and clinical data.

### 3.2. Results of HADS, AIS, ACE-III, and MoCA for the Entire Sample (Without Stratification by Intervention Type or Setting)

Following the application of relaxation techniques, patients showed improvement in anxiety and depressive symptoms, with the reduction in anxiety severity being more pronounced than that in depressive symptoms. A decrease in the mean AIS total score by more than 3.5 points was also observed, indicating improved sleep quality among patients undergoing relaxation. Detailed data are presented in Table 2.

With respect to cognitive functions, the interventions contributed to an overall improvement as assessed by the MoCA and ACE-III (total scores), although changes within individual cognitive domains did not reach statistical significance. Detailed results are presented in Table 3.

### 3.3. Results of HADS, AIS, ACE-III, and MoCA for Standard Group Relaxation Using the Schultz Method in the Inpatient Setting

In the group undergoing standard group relaxation using the Schultz method, a reduction in anxiety level measured by the HADS and a marked improvement in sleep quality were observed. The mean severity of depressive symptoms also decreased, but this parameter did not reach statistical significance. Detailed data are presented in Table 4.

No statistically significant change was observed in cognitive functions. Detailed data are presented in Table 5.

### 3.4. Results of HADS, AIS, ACE-III, and MoCA for Standard Relaxation Using the Schultz Method in the Home Setting

In the group performing standard relaxation using the Schultz technique in the home setting, no statistically significant changes were observed in parameters related to anxiety, depression, or sleep quality. Detailed data are presented in Table 6.

In the group of female patients performing standard relaxation in the home setting, the intervention had a positive effect on overall cognitive performance as measured by the ACE-III; however, no significant changes were observed in individual cognitive domains or on the MoCA. Detailed data are presented in Table 7.

### 3.5. Results of HADS, AIS, ACE-III, and MoCA for Group VR-Based Relaxation in the Inpatient Setting

Among patients undergoing group VR-based relaxation in the inpatient setting, reductions in depressive and anxiety symptoms were observed, along with improved sleep quality. Detailed data are presented in Table 8.

No statistically significant change was observed in cognitive functions. Detailed data are presented in Table 9.

### 3.6. Results for VR-Based Relaxation in the Home Setting

For the group of female patients using VR-based relaxation in the home setting, a statistically significant improvement in sleep quality was obtained. Detailed data are presented in Table 10.

No statistically significant change was observed in cognitive functions. Detailed data are presented in Table 11.

A summary of the HADS, AIS, MoCA and ACE-III scores before and after the intervention for each patient group is presented in Table 12.

### 3.7. Safety and Adverse Events

Throughout the study, no adverse events related to either autogenic training or VR-based relaxation were observed. None of the participants reported nausea, dizziness, increased anxiety, headache, or cybersickness during or after the sessions. No serious adverse events requiring medical intervention occurred, and no participant discontinued the study for safety-related reasons. 

## 4. Discussion

In this study, the primary endpoint was the change in the severity of anxiety symptoms and sleep quality, assessed using the HADS-A and AIS. The observed effects, a 2–6-point reduction on HADS-A and a 2–4-point reduction on AIS, fall within the range considered clinically meaningful in the literature (minimal clinically important difference, MCID), typically around 1.5–2 points for HADS-A and 3 points for AIS. Although the confidence intervals remain relatively wide due to the limited sample size, the consistent direction of the effects indicates a potentially beneficial impact of both relaxation methods, particularly in inpatient settings, and provides justification for conducting a confirmatory study with greater statistical power. In parallel with these findings, recent technological advances have led to a substantial increase in interest in the use of VR in the diagnosis and treatment of mental disorders. Owing to its ability to create a safe and controlled environment, VR is particularly valuable in exposure therapy for social phobia [36,37], specific phobias [38,39,40], post-traumatic stress disorder [41], obsessive–compulsive disorder [42], and even addictions [43]. Against this backdrop, the present study aimed to demonstrate an alternative application of VR as an adjunctive tool in relaxation therapy and to compare its effectiveness with traditional Schultz autogenic training. 

### 4.1. Cognitive Functions

Analysis of the entire cohort showed an overall improvement in cognitive functions (*p* = 0.046 for MoCA and *p* = 0.012 for ACE-III); however, a more detailed analysis reveals that a statistically significant improvement (*p* = 0.013) was obtained only in the group using Schultz autogenic training for relaxation in the home setting. This may be attributable to the therapeutic intervention being too brief to induce lasting changes in patients’ cognitive functioning. It is also noteworthy that the baseline level of cognitive functions among participants was high, which may have limited the potential for further improvement. Although no statistically significant effect of VR-based therapy on cognitive functions was observed in the other groups of female patients in this study, this issue undoubtedly warrants further exploration, particularly among patients with cognitive impairment, for whom, according to the literature [44], VR-based relaxation yields promising results. 

### 4.2. Insomnia

Schultz autogenic training in the inpatient setting showed the largest effect among the relaxation methods examined with respect to improving sleep quality (*p* = 0.012 for AIS), which may be of clinical significance as a non-pharmacological intervention for insomnia. For VR-based therapy in both hospital and home settings, a statistically significant improvement in sleep quality was also observed (*p* = 0.002 and *p* = 0.034, respectively, for AIS); however, this effect was smaller than that of Schultz autogenic training under the same conditions. These data are consistent with studies suggesting a positive impact of Schultz autogenic training on sleep (a greater sense of freshness and energy upon awakening, faster return to sleep after nocturnal awakenings) [45]. The impact of VR on sleep quality requires further investigation; nonetheless, the results obtained in this study, as well as prior analyses [21], indicate the potential of VR in the treatment of insomnia. VR-based relaxation may favorably influence not only subjective sleep assessment but also more objective parameters such as deep sleep duration and sleep efficiency [46].

### 4.3. Anxiety Symptoms

Both Schultz autogenic training and VR-based therapy in the inpatient setting had a statistically significant effect on reducing anxiety symptoms (*p* < 0.01 and *p* < 0.001, respectively, for HADS-Anxiety). No statistically significant improvement was observed in the remaining groups. These results are consistent with prior studies demonstrating that VR-based relaxation alleviates anxiety and worry; moreover, patients using this form of therapy showed greater engagement and a higher completion rate of relaxation sessions compared with the control group [24]. VR-based relaxation enables full immersion in a virtual environment and near-immediate sensory stimulation, and it does not require prior training or skills, whereas autogenic training requires systematic practice and active concentration, which can be discouraging at the outset and may contribute to discontinuation of subsequent therapeutic sessions. Analyses to date on the impact of autogenic training on anxiety levels align with our findings; the role of autogenic training in regulating vital parameters such as blood pressure [47] and heart rate variability [48] is also emphasized, which may be relevant in the treatment of conditions presenting with somatic symptoms.

### 4.4. Depressive Symptoms

Among the relaxation methods examined, a statistically significant reduction in depressive symptoms was observed in the group of female patients undergoing group relaxation using VR headsets in the inpatient setting (mean decrease of 4.00 points, *p* = 0.035 for HADS-Depression). In the remaining groups, although a similar trend was noted, the data did not reach statistical significance. There is a paucity of studies that unambiguously confirm the effect of VR-based therapy on the treatment of depression; however, preliminary observations suggest the potential efficacy of this relaxation modality. The available literature indicates that integrating VR technology with cognitive–behavioral therapy, through an immersive experience, may yield benefits in reducing suicide risk, increase patient engagement and treatment adherence, and be comparable to pharmacotherapy in terms of reducing depressive symptoms [49].

### 4.5. Influence of the Relaxation Environment on Effectiveness

The data indicate that the environment in which patients engage in relaxation therapy also significantly affects its outcomes. Both Schultz autogenic training and VR-based relaxation proved more effective in the inpatient setting than at home; only with respect to improvement in cognitive functions did the home environment turn out to be the better setting for therapeutic intervention. The lower effectiveness of home-based relaxation may be attributable to the presence of more distractors than in the controlled ward environment (e.g., noise, other household members) and the absence of a therapist supervising the session, which may be associated with lower motivation and reduced patient engagement. Group relaxation creates a sense of community and support, which may likewise translate into greater effectiveness. 

Existing research on VR-based relaxation indicates that immersive virtual environments can reduce anxiety and tension by combining sensory distraction with emotion regulation [50,51]. The effects observed in the present project, particularly in inpatient settings, align with previous findings in which VR proved more effective than standard audio-based relaxation procedures. 

With regard to autogenic training, our results are consistent with the effect profiles reported in the meta-analyses by Stetter and Kupper (2002) and in the studies by Kanji et al. (2006), where AT led to reductions in anxiety symptoms and improvements in sleep, although it required stable conditions and sufficient patient engagement [12,47]. 

Differences between our findings and earlier publications may stem from varying levels of immersion, a more clinically heterogeneous population, and differences in the settings in which the interventions were conducted. 

According to current literature, immersion—defined as the set of technological properties of a VR system—influences the strength of the presence experience, understood as the subjective feeling of “being” in the virtual environment [52,53]. Presence is considered one of the key mechanisms underlying symptom reduction because it facilitates deeper cognitive and emotional engagement, which in turn supports arousal regulation and distraction from stressors [20,54]. 

Although immersion and presence were not measured in the present study, differences between the VR outcomes in inpatient and home settings may indicate the relevance of these mechanisms. In a controlled environment with fewer distracting stimuli, technological immersion may yield a stronger sense of presence, which, according to previous research, is associated with greater reductions in anxiety and stress [55]. 

The differences in the effectiveness of VR and AT in hospital versus home settings are consistent with literature on the impact of therapeutic context on the efficacy of psychological interventions. Craske et al. (2014) and Bakker and Kazantzis (2016) indicate that controlled environments promote greater focus, reduce the number of distractors, and increase adherence to instructions, which translates into stronger therapeutic effects [14,15]. In home settings, where distracting stimuli and reduced motivation are more likely, intervention effectiveness tends to be lower, which is reflected in our results. At the same time, the VR effects observed in the home setting remain consistent with previous reports suggesting that even a moderate level of immersion can positively influence sleep quality and subjective relaxation [56]. 

The findings of this project can also be interpreted in light of research on emotion regulation and sensory distraction. Gross’s models (2015) emphasize that effective emotion regulation depends on the ability to shift attentional focus and modulate arousal [57]. VR, through multisensory stimulation, may function as a strong emotion regulator by reducing sympathetic nervous system activity and facilitating entry into a relaxed state. This effect has been repeatedly observed in studies on pain [50] and stress [58], providing a coherent explanation for the changes in anxiety and sleep observed in our study. 

### 4.6. Limitations and Opportunities

This study is a pilot; accordingly, the aim was a preliminary analysis of the effects of the therapeutic interventions. Nevertheless, the findings are promising and provide a starting point for further exploration. To our knowledge, this is the first comparative analysis to assess the impact of relaxation via autogenic training and VR headsets on sleep quality, reduction of depressive and anxiety symptoms, and cognitive functions in both inpatient and outpatient populations. Limitations include the small size of the study groups and the short duration of the intervention, which hampers assessment of the durability of the relaxation effects. Some patients did not complete the required number of sessions due to a brief a hospitalization or to a deterioration in mental state that precluded continuation of the experiment. Moreover, the severity of depressive symptoms, anxiety, and sleep disturbances was assessed using self-report instruments, which may also affect the accuracy of the results obtained. Cognitive functions were assessed with brief screening instruments (MoCA, ACE-III) administered twice within a short, two-week interval. The small improvements observed on these measures should therefore be interpreted with caution, as they may partly reflect practice or retest effects rather than true intervention-related change in cognitive functioning. 

Based on the findings, it can be preliminarily concluded that VR-based relaxation is an effective adjunctive therapeutic tool supporting the treatment of insomnia as well as depressive and anxiety disorders. The development of modern VR technology and efforts to use it in the diagnosis and treatment of mental disorders open new possibilities for patients for whom traditional relaxation methods have not yielded the desired effects. Relaxation using VR headsets may be particularly helpful for individuals with limited mobility or in poor health—including mental health—that impedes social activity and in-person meetings with a therapist. As demonstrated by the COVID-19 pandemic, this technology can also serve as support in situations requiring social isolation [59]. Patients staying on long-term care wards, for whom the hospital environment is an everyday reality, may likewise benefit from VR-based relaxation. Full immersion in a virtual world and focusing attention on pleasant stimuli allow patients to momentarily forget where they are and divert attention from other unpleasant stimuli, such as pain [60]. 

Digital exclusion, however, remains a problem especially among groups with lower socioeconomic status and older patients [61] which hinders large-scale implementation of this form of relaxation therapy. Nevertheless, conducting further research on the use of VR in the treatment and diagnosis of mental disorders appears both necessary and promising, and the rationale for relaxation via Schultz autogenic training remains current. 

In terms of limitations, it should also be noted that the study included only women, which hinders the generalization of the results to male populations. Sex differences in stress reactivity and sensitivity to emotional stimuli are well documented [62,63] and may influence responses to both VR and autogenic training. Moreover, although diagnostic heterogeneity increases the ecological validity of the study, it limits the ability to precisely identify the groups that might benefit most from each form of relaxation. 

The pilot nature of this study is closely linked to the capacity and profile of the recruiting site. The trial was conducted in a single 25-bed female psychiatric ward, which inherently constrained recruitment and resulted in relatively small groups in each arm. To maintain a clinically clean sample and reduce diagnostic heterogeneity, we included only female patients with psychiatric diagnoses confirmed by a full psychiatric assessment and standardized psychological testing. While these design choices increased internal validity and ensured more reliable between-group comparisons by keeping the sizes of the remaining groups proportionate to the inpatient ward group, they also limited statistical power and restrict the generalizability to more heterogeneous clinical populations. 

Based on the results of the present project, a confirmatory study could be designed with a simplified two-arm structure (VR vs. AT) conducted in inpatient settings, where the strongest effects were observed. Assuming a conservative variance (SD ≈ 4) and a clinically meaningful effect of 2–3 points on the HADS-A and AIS, a sample of 60–80 participants would be required to achieve a power of 0.80. The study should include systematic measurement of immersion and presence (e.g., ITC-SOPI or the Slater–Usoh–Steed questionnaire), which would allow for mediation analysis to determine whether presence mediates the impact of VR on symptom reduction. Enhancing the intervention with more immersive environments (interactive scenarios, wider field of view, biofeedback components) would additionally make it possible to assess whether increasing technological parameters meaningfully amplifies clinical effects. 

## 5. Conclusions

1. VR-based relaxation in the inpatient setting was more effective than at home, particularly in improving sleep quality and reducing anxiety levels, which may have resulted from fewer distractors, the presence of a therapist, and social support.

2. Group VR relaxation delivered in the inpatient setting proved more effective than Schultz autogenic training conducted in the same environment, especially in reducing anxiety severity and improving sleep quality. By contrast, the effects of both methods on cognitive functions in the inpatient setting did not reach statistical significance.

3. Home-based relaxation using VR technology showed greater efficacy in improving sleep quality, whereas a statistically significant improvement in cognitive functions was observed with autogenic training.

4. A statistically significant improvement in cognitive functions was observed only in the group using Schultz autogenic training in the home setting.

## Figures and Tables

**Table 1:** Characteristics of sociodemographic and clinical data.

Age Range	Standard Relaxation with Schultz Autogenic Training (*n* = 20)	Relaxation with VR Headsets (*n* = 17)
18–24	6 (30%)	5 (29.41%)
25–34	3 (15%)	4 (23.53%)
35–54	2 (10%)	5 (29.41%)
55–64	6 (30%)	0
65+	3 (15%)	3 (17.65%)
**Education**		
Primary	0	2 (11.76%)
Vocational	8 (40%)	1 (5.88%)
Secondary	9 (45%)	5 (29.41%)
Higher	3 (15%)	9 (52.94%)
**Diagnosis**		
Depressive disorder	9 (45%)	9 (52.94%)
Mixed anxiety–depressive disorder	8 (40%)	3 (17.65%)
Bipolar affective disorder	1 (5%)	2 (11.76%)
Anxiety disorders	2 (10%)	2 (11.76%)
Adjustment disorders	0	1 (5.88%)

**Table 2:** Results of HADS and AIS before and after the intervention for the entire group of female patients (Student’s *t*-test *p* < 0.05).

Scale	Before, Mean (SD)	After, Mean (SD)	Difference			
			*t*	−95% CI	+95% CI	*p* Value
HADS—anxiety	12.89 (4.79)	10.05 (4.30)	5.31	1.75	3.92	0.000006
HADS—depression	10.14 (5.00)	8.22 (5.04)	3.06	0.65	3.19	0.004196
AIS	10.03 (4.75)	6.41 (3.67)	5.10	2.18	5.06	0.000011

**Table 3:** Results of MoCA and ACE-III before and after the intervention for the entire group of female patients (sign test *p* < 0.05).

Scale	Before, Mean (SD)	After, Mean (SD)	Difference	
			Z	*p* Value
MOCA	25.84 (4,78)	26.86 (3.71)	2.00	0.045500
ACE-III—Attention	16.95 (1.68)	17.24 (1.62)	1.55	0.121335
ACE-III—Memory	21.92 (5.67)	23.57 (3.48)	1.92	0.055009
ACE-III—Fluency	12.14 (2.63)	12.41 (2.31)	0.67	0.502335
ACE-III—Language	25.11 (2.20)	25.62 (1.06)	1.81	0.070440
ACE-III—Visuospatial functions	14.30 (1.96)	14.35 (2.03)	0.00	1.000000
ACE-III Total score	90.41 (11.78)	93.19 (8.31)	2.51	0.011921

**Table 4:** Results of HADS and AIS before and after the intervention for the group of female patients undergoing standard group relaxation using the Schultz method (Student’s *t*-test *p* < 0.05).

Scale	Before, Mean (SD)	After, Mean (SD)	Difference			
			*t*	−95% CI	+95% CI	*p* Value
HADS—anxiety	16.00 (2.62)	12.00 (3.89)	3.49	1.41	6.60	0.006794
HADS—depression	14.00 (3.16)	11.40 (4.45)	1.77	0.72	5.92	0.110668
AIS	13.7 (5.50)	7.50 (4.28)	3.15	1.74	10.74	0.011818

**Table 5:** Results of MoCA and ACE-III before and after the intervention for the group of female patients undergoing standard group relaxation using the Schultz method (sign test *p* < 0.05).

Scale	Before, Mean (SD)	After, Mean (SD)	Difference	
			Z	*p* Value
MOCA	21.90 (5.90)	23.90 (5.22)	0.677	0.504
ACE-III—Attention	16.10 (2.08)	16.30 (2.36)	0.408	0.683
ACE-III—Memory	18.00 (7.15)	20.80 (4.57)	0.756	0.450
ACE-III—Fluency	11.10 (3.45)	11.10 (2.47)	0.00	1.00
ACE-III—Language	24.90 (1.85)	25.10 (1.52)	0.894	0.371
ACE-III—Visuospatial functions	13.50 (2.64)	12.50 (2.59)	1.512	0.131
ACE-III Total score	83.60 (14.68)	85.80 (10.05)	−0.316	0.752

**Table 6:** Results of HADS and AIS before and after the intervention for the group of female patients performing standard relaxation using the Schultz technique in the home setting (Student’s *t*-test *p* < 0.05).

Scale	Before, Mean (SD)	After, Mean (SD)	Difference			
			*t*	−95% CI	+95% CI	*p* Value
HADS—anxiety	10.00 (4.94)	9.30 (4.40)	0.82	−1.24	2.64	0.435
HADS—depression	7.00 (4.85)	6.90 (4.09)	0.09	−2.37	2.57	0.929
AIS	8.20 (3.85)	6.70 (4.37)	1.48	−0.79	3.79	0.173

**Table 7:** Results of MoCA and ACE-III before and after the intervention for the group of female patients performing standard relaxation using the Schultz technique in the home setting (sign test *p* < 0.05).

Scale	Before, Mean (SD)	After, Mean (SD)	Difference	
			Z	*p* Value
MOCA	29.10 (0.99)	29.50 (0.71)	0.894	0.371
ACE-III—Attention	17.10 (1.37)	17.60 (1.26)	1.155	0.248
ACE-III—Memory	25.80 (0.42)	26.00 (0.00)	0.707	0.480
ACE-III—Fluency	13.20 (0.79)	13.60 (0.70)	1.50	0.134
ACE-III—Language	26.00 (0.00)	26.00 (0.00)		
ACE-III—Visuospatial functions	15.20 (1.23)	15.60 (0.70)	0.00	1.00
ACE-III Total score	97.30 (1.89)	98.80 (1.40)	2.475	0.013

**Table 8:** Results of HADS and AIS before and after the intervention for the group of female patients undergoing group relaxation using VR headsets (Student’s *t*-test *p* < 0.05).

Scale	Before, Mean (SD)	After, Mean (SD)	Difference			
			*t*	−95% CI	+95% CI	*p* Value
HADS—anxiety	14.60 (4.50)	10.30 (4.95)	5.88	2.65	5.95	<0.001
HADS—depression	12.30 (3.16)	9.20 (5.49)	2.48	0.27	5.93	0.035
AIS	9.60 (3.75)	5.80 (3.08)	4.32	1.81	5.79	0.002

**Table 9:** Results of MoCA and ACE-III before and after the intervention for the group of female patients undergoing group relaxation using VR headsets (sign test *p* < 0.05).

Scale	Before, Mean (SD)	After, Mean (SD)	Difference	
			Z	*p* Value
MOCA	25.40 (4.48)	26.30 (2.50)	0.756	0.450
ACE-III—Attention	17.10 (1.91)	17.50 (1.27)	0.500	0.617
ACE-III—Memory	20.30 (5.85)	23.00 (2.94)	0.667	0.505
ACE-III—Fluency	11.90 (3.18)	11.90 (3.18)	−0.408	0.683
ACE-III—Language	23.80 (3.52)	25.50 (1.27)	1.225	0.221
ACE-III—Visuospatial functions	13.80 (1.81)	14.60 (0.97)	1.225	0.221
ACE-III Total score	86.90 (13.76)	92.50 (7.53)	0.667	0.505

**Table 10:** Results of HADS and AIS before and after the intervention for the group of female patients using VR-based relaxation in the home setting (Student’s *t*-test *p* < 0.05).

Scale	Before, Mean (SD)	After, Mean (SD)	Difference			
			*t*	−95% CI	+95% CI	*p* Value
HADS—anxiety	10.14 (4.10)	8.00 (3.21)	1.72	−0.90	5.18	0.135
HADS—depression	6.00 (3.92)	4.14 (3.39)	2.17	−0.24	3.95	0.073
AIS	8.00 (3.70)	5.29 (2.50)	2.73	0.28	5.14	0.034

**Table 11:** Results of MoCA and ACE-III before and after the intervention for the group of female patients using VR-based relaxation in the home setting (sign test *p* < 0.05).

Scale	Before, Mean (SD)	After, Mean (SD)	Difference	
			Z	*p* Value
MOCA	27.43 (2.57)	28.14 (1.77)	0.500	0.617
ACE-III—Attention	17.71 (0.49)	17.71 (0.76)	−0.707	0.480
ACE-III—Memory	24.29 (1.70)	24.86 (1.77)	0.500	0.617
ACE-III—Fluency	12.43 (1.90)	13.29 (0.76)	0	1
ACE-III—Language	26.00 (0.00)	26.00 (0.00)		
ACE-III—Visuospatial functions	14.86 (1.46)	14.86 (1.95)	0	1
ACE-III Total score	95.29 (3.94)	96.71 (3.95)	1.50	0.134

**Table 12:** Summary table of HADS, AIS, MoCA, and ACE-III results before and after the intervention for all patient groups.

Scale		HADS—Anxiety	HADS—Depression	AIS	MOCA	ACE-III
Standard group relaxation using the Schultz method	Before, mean	16	14	13.7	21.9	83.6
	After, mean	12	11.4	7.5	23.9	85.8
	*p*-value	0.007	0.111	0.012	0.504	0.752
Standard relaxation using the Schultz method in the home setting	Before, mean	10	7	8.2	29.1	97.3
	After, mean	9.3	6.9	6.7	29.5	98.8
	*p*-value	0.435	0.929	0.173	0.371	0.013
Group relaxation using VR headsets	Before, mean	14.6	12.3	9.6	25.4	86.9
	After, mean	10.3	9.2	5.8	26.3	92.5
	*p*-value	<0.001	0.035	0.002	0.45	0.505
Relaxation using VR headsets in the home setting	Before, mean	10.14	6	8	27.43	95.29
	After, mean	8	4.14	5.29	28.14	96.71
	*p*-value	0.135	0.073	0.034	0.617	0.134

## Data Availability

The data that supports the findings of the manuscript may be available from the corresponding author upon reasonable request.

## Supplementary Materials

The following supporting information can be downloaded at: https://www.mdpi.com/article/10.3390/reports8040247/s1, Relaxation script in Polish; Relaxation script in English; Technical specification; Table S1: Parameters of the VR session; Session description; Table S2: Descriptive statistics for the whole study sample; Figure S1: HADS anxiety scores before and after the interventions in the whole study sample (*n* = 37); Figure S2: HADS depression scores before and after the interventions in the whole study sample (*n* = 37); Figure S3: AIS scores before and after the interventions in the whole study sample (*n* = 37); Figure S4: MOCA scores before and after the interventions in the whole study sample (*n* = 37); Figure S5: ACE-III scores before and after the interventions in the whole study sample (*n* = 37).

## Abbreviations

The following abbreviations are used in this manuscript:

VR	Virtual reality
AT	Autogenic training
WHO	World Health Organization
ICD-11	International Classification of Diseases, 11th Revision
TMS	Transcranial magnetic stimulation
tDCS	Transcranial direct current stimulation
HADS	Hospital Anxiety and Depression Scale
HADS-A	Hospital Anxiety and Depression Scale—Anxiety subscale
AIS	Athens Insomnia Scale
ACE-III	Addenbrooke’s Cognitive Examination III
MoCA	Montreal Cognitive Assessment
SD	Standard deviation
CI	Confidence interval
MCID	Minimal clinically important difference
ITC-SOPI	ITC-Sense of Presence Inventory
