# Supplementary material for: Use of Virtual Reality (VR)-Based Relaxation Among Female Patients with Mental Disorders: A Pilot Study
Source: Reports (MDPI). 2025 Nov 26;8(4):247. doi: 10.3390/reports8040247 (PMC12736646; doi:10.3390/reports8040247)

`

Relaxation script in Polish

Witaj! To nagranie przeprowadzi cię przez relaksację autogenną, w której połączymy techniki medytacji i autosugestii. Metoda ta pozwala dzięki mocy słów wywołać w naszym organizmie głęboką relaksację, wyciszenie i poczucie senności.

A teraz proszę: zamknij oczy, usiądź wygodnie na krześle lub połóż się w jak najwygodniejszej pozycji. Najlepiej, jeśli położysz się na plecach, z rękoma ułożonymi wzdłuż ciała i nogami w swobodnym rozkroku. Jeśli nie chcesz się kłaść, po prostu usiądź na wygodnym fotelu, z rękoma swobodnie ułożonymi i stopami równomiernie dotykającymi podłoża.

Jesteś już w wygodnej pozycji. Pozwól, by twoje oczy delikatnie się zamknęły. Zacznij od przeniesienia uwagi na oddech. Wdychaj powietrze nosem i wydychaj ustami lub nosem — jak ci wygodniej. Poczuj chłodne powietrze, które trafia do twojego ciała wraz z wdechem, i nieco cieplejsze, które opuszcza twoje ciało przy wydechu. Podczas wdechu wyobraź sobie, że zaczyna się on na samym czubku twojej głowy, a podczas wydechu przechodzi przez całe twoje ciało — od głowy do stóp. Niech wdech będzie głęboki, wolny i gładki. Podczas tej relaksacji będziemy skupiać się na różnych odczuciach ciała. Twoim zadaniem jest tylko powtarzanie w myślach moich instrukcji i skupienie się na odczuciach własnego ciała. Nie musisz robić nic więcej — pozwól, by ta relaksacja po prostu działała sama. Nie próbuj niczego przyspieszać ani niczego wymuszać — podążaj tylko za moim głosem.

A teraz przenieś uwagę na całe swoje ciało. Zauważ odczucia, jakie z niego płyną. Czy czujesz swój puls, bicie serca, może delikatne napięcie, ciężkość? Obserwuj przez chwilę.

A teraz, powtarzając w myślach wypowiadane przeze mnie słowa, skup się na wrażeniach płynących z ciała. Skup uwagę na prawej ręce i w myślach powtórz: czuję spokój i odprężenie. Moja prawa ręka jest ciężka. Moja prawa ręka jest ciężka. Moja prawa ręka jest ciężka. Moja prawa ręka jest ciężka. Moja prawa ręka jest ciężka.

Przenieś teraz uwagę na swoją lewą rękę i powtarzaj w myślach za mną: moja lewa ręka jest ciężka. Moja lewa ręka jest ciężka. Moja lewa ręka jest ciężka. Moja lewa ręka jest ciężka. Moja lewa ręka jest ciężka.

Poczuj głęboką ciężkość i obejmij uwagą swoje obie ręce, powtarzając w myślach: czuję spokój i odprężenie. Moje obie ręce są ciężkie. Moje obie ręce są ciężkie. Moje obie ręce są ciężkie. Moje obie ręce są ciężkie.

Przenieś teraz swoją uwagę na prawą nogę i powtarzaj: czuję spokój i odprężenie. Moja prawa noga jest ciężka. Moja prawa noga jest ciężka. Moja prawa noga jest ciężka. Moja prawa noga jest ciężka. Moja prawa noga jest ciężka.

Obserwuj odczucia jeszcze przez chwilę i przenieś uwagę na lewą nogę: czuję spokój i odprężenie. Moja lewa noga jest ciężka. Moja lewa noga jest ciężka. Moja lewa noga jest ciężka. Moja lewa noga jest ciężka. Moja lewa noga jest ciężka.

Obejmij teraz uwagą obie swoje nogi i powtarzaj za mną: czuję spokój i odprężenie. Moje obie nogi są ciężkie. Moje obie nogi są ciężkie. Moje obie nogi są ciężkie. Moje obie nogi są ciężkie. Moje obie nogi są ciężkie.

Wyobraź sobie, że leżysz teraz na najbardziej miękkim materiale na świecie, a ciężar nóg i rąk sprawia, że delikatnie się zapadasz, zatapiasz w spokoju, ciszy i relaksacji. Czujesz spokój. Obejmij świadomością swoje obie ręce i nogi, obejmując odczucia, które w nich są, i powtarzaj w myślach: czuję spokój i odprężenie. Moje nogi i ręce są ciężkie. Moje nogi i ręce są ciężkie. Moje nogi i ręce są ciężkie. Moje nogi i ręce są ciężkie. Moje nogi i ręce są ciężkie.

Poczuj, jak całe ciało staje się całkowicie odprężone i delikatnie senne. Teraz skupimy się na odczuciach przyjemnego, kojącego ciepła, które sprawia, że twoje mięśnie stają się jeszcze bardziej rozluźnione. Wróć jeszcze raz uwagą do prawej ręki i powtarzaj w myślach za mną: czuję spokój i odprężenie. Moja prawa ręka jest ciężka i ciepła. Moja prawa ręka jest ciężka i ciepła. Moja prawa ręka jest ciężka i ciepła. Moja prawa ręka jest ciężka i ciepła. Moja prawa ręka jest ciężka i ciepła. Moja prawa ręka jest ciężka i ciepła.

`

Poczuj pogłębiające się, przyjemne uczucia w prawej ręce i bardzo powoli skup się na lewej. W myślach powtarzaj: czuję spokój i odprężenie. Moja lewa ręka jest ciężka i ciepła. Moja lewa ręka jest ciężka i ciepła. Moja lewa ręka jest ciężka i ciepła. Moja lewa ręka jest ciężka i ciepła. Moja lewa ręka jest ciężka i ciepła.

Obserwuj przez moment odczucia w lewej ręce. Czuję spokój i odprężenie. Obejmij uwagą teraz obydwie dłonie i ręce i w myślach powtarzaj: moje ręce są ciepłe i ciężkie. Moje ręce są ciepłe i ciężkie. Moje ręce są ciepłe i ciężkie. Moje ręce są ciepłe i ciężkie. Moje ręce są ciepłe i ciężkie.

Poczuj, jak twoje ręce wytwarzają ciepłą, relaksującą falę, która oblewa całe twoje ciało. Skup się teraz na prawej nodze i w myślach powtarzaj: czuję spokój i odprężenie. Moja prawa noga jest ciężka i ciepła. Moja prawa noga jest ciężka i ciepła. Moja prawa noga jest ciężka i ciepła. Moja prawa noga jest ciężka i ciepła. Moja prawa noga jest ciężka i ciepła.

Zauważ jeszcze przez kilka sekund odczucia płynące z prawej nogi i łagodnie, powoli przenieś uwagę na lewą nogę, powtarzając za mną w myślach: czuję spokój i odprężenie. Moja lewa noga jest ciężka i ciepła. Moja lewa noga jest ciężka i ciepła. Moja lewa noga jest ciężka i ciepła. Moja lewa noga jest ciężka i ciepła. Moja lewa noga jest ciężka i ciepła.

Obejmij swoją uwagą obie nogi i powtarzaj za mną: czuję spokój i odprężenie. Moje obie nogi są ciężkie i ciepłe. Moje obie nogi są ciężkie i ciepłe. Moje obie nogi są ciężkie i ciepłe. Moje obie nogi są ciężkie i ciepłe. Moje obie nogi są ciężkie i ciepłe.

Przenieś swoją uwagę na całe swoje ręce i nogi i powtarzaj w myślach: czuję spokój i odprężenie. Zauważ poczucie ciepła i ciężkości w całym ciele. Obserwuj, czy zmienia się natężenie tych odczuć — może jakaś część ciała jest wyjątkowo ciepła, a któraś bardziej ciężka niż inna. Obserwuj swoje ciało, nie próbując zmieniać tego, co czujesz. Czuję spokój i odprężenie. Poczuj, jak twoje ciało staje się całkowicie odprężone i delikatnie senne.

Wsłuchaj się w swoje serce i powtarzaj: czuję spokój i rozluźnienie. Powtarzaj w myślach: moje serce bije spokojnie i równomiernie. Moje serce bije spokojnie i równomiernie. Moje serce bije spokojnie i równomiernie. Moje serce bije spokojnie i równomiernie. Moje serce bije spokojnie i równomiernie.

A teraz przenieś uwagę odrobinę wyżej, na swoją głowę, i poczuj, jak twoje czoło oblewa przyjemny chłód. W myślach powtarzaj: moje czoło jest chłodne i czuję rozluźnienie. Moje czoło jest chłodne i czuję rozluźnienie. Moje czoło jest chłodne i czuję rozluźnienie. Moje czoło jest chłodne i czuję rozluźnienie. Moje czoło jest chłodne i czuję rozluźnienie.

Zauważ, jak różne odczucia mogą dać ci poczucie głębokiego spokoju i odprężenia. Teraz zwróć uwagę na to, co czujesz w swoim ciele, zaczynając od głowy do nóg. Powtarzaj w myślach za mną: moje czoło jest chłodne i czuję rozluźnienie. Moje czoło jest chłodne i czuję rozluźnienie. Moje serce bije spokojnie i równomiernie. Moje serce bije spokojnie i równomiernie. Mój brzuch jest przyjemnie ciepły i zrelaksowany. Mój brzuch jest przyjemnie ciepły i zrelaksowany. Moje ręce są ciężkie i ciepłe. Moje ręce są ciężkie i ciepłe. Moje nogi są ciężkie i ciepłe. Moje nogi są ciężkie i ciepłe.

Zanurz się w tych wszystkich przyjemnych odczuciach.

W ten oto sposób dobiegliśmy do końca naszej dzisiejszej praktyki medytacji. Mam nadzieję, że to nagranie pomogło ci się rozluźnić i uspokoić. Ale proszę, pamiętaj: techniki relaksacyjne i medytacja to praktyka — częste powtarzanie sprawia, że stajemy się w tym coraz lepsi, a dzięki temu lepiej kontrolujemy nasz organizm i umysł. Jeśli dziś nie czujesz wyraźnej zmiany, daj sobie czas. Nie przyspieszaj niczego — w pewnym momencie zaskoczysz się, jak mocno mogą zmienić się twoje odczucia. A teraz dziękuję ci i do zobaczenia następnym razem.

Relaxation script in English

Wel-come! This recording will guide you through autogenic relaxation, combining techniques of meditation and autosuggestion. By harnessing the power of words, this method elicits deep relaxation, calm, and a gentle sense of drowsiness.

Now, please close your eyes and sit comfortably on a chair or lie down in the most comfortable position. Ideally, lie on your back with your arms alongside your body and your legs resting slightly

apart. If you prefer not to lie down, simply sit in a comfortable armchair with your arms resting freely and your feet evenly supported on the floor.

You are now in a comfortable position. Allow your eyes to close softly. Begin by bringing your attention to your breath. Inhale through your nose and exhale through your mouth or nose—whichever feels more natural. Notice the cool air entering your body with each inhale and the slightly warmer air leaving with each exhale. As you inhale, imagine the breath beginning at the very crown of your head; as you exhale, imagine it flowing through your whole body—from head to toes. Let each breath be deep, slow, and smooth. During this relaxation, we will focus on bodily sensations. Your only task is to repeat my instructions silently and attend to the sensations in your body. You do not need to do anything else—allow the process to unfold on its own. Do not try to hurry or force anything—simply follow my voice.

Now shift your attention to your whole body. Notice the sensations arising—your pulse, the beating of your heart, perhaps a subtle tension or a sense of heaviness. Observe for a moment.

Next, repeating my words silently, focus on the sensations in your body. Bring your attention to your right arm and repeat in your mind: I feel calm and relaxed. My right arm is heavy. My right arm is heavy. My right arm is heavy. My right arm is heavy. My right arm is heavy.

Move your attention to your left arm and repeat silently after me: My left arm is heavy. My left arm is heavy. My left arm is heavy. My left arm is heavy. My left arm is heavy.

Sense a deep heaviness and include both arms in your awareness, repeating: I feel calm and relaxed. Both of my arms are heavy. Both of my arms are heavy. Both of my arms are heavy. Both of my arms are heavy.

Now shift your attention to your right leg and repeat: I feel calm and relaxed. My right leg is heavy. My right leg is heavy. My right leg is heavy. My right leg is heavy. My right leg is heavy.

Observe the sensations for a moment, then move your attention to your left leg: I feel calm and relaxed. My left leg is heavy. My left leg is heavy. My left leg is heavy. My left leg is heavy. My left leg is heavy.

Now include both legs in your awareness and repeat after me: I feel calm and relaxed. Both of my legs are heavy. Both of my legs are heavy. Both of my legs are heavy. Both of my legs are heavy. Both of my legs are heavy.

Imagine you are lying on the softest surface in the world, and the weight of your legs and arms allows you to gently sink in—into quiet, peace, and relaxation. You feel calm. Encompass both your arms and legs with your awareness, noticing the sensations within them, and repeat silently: I feel calm and relaxed. My legs and arms are heavy. My legs and arms are heavy. My legs and arms are heavy. My legs and arms are heavy. My legs and arms are heavy.

Feel your whole body becoming fully relaxed and gently drowsy. We will now focus on the sensation of pleasant, soothing warmth that allows your muscles to loosen even more. Re-turn your attention to your right arm and repeat silently after me: I feel calm and relaxed. My right arm is heavy and warm. My right arm is heavy and warm. My right arm is heavy and warm. My right arm is heavy and warm. My right arm is heavy and warm. My right arm is heavy and warm.

Notice the deepening, pleasant sensations in the right arm and, very slowly, shift your attention to the left arm. Repeat in your mind: I feel calm and relaxed. My left arm is heavy and warm. My left arm is heavy and warm. My left arm is heavy and warm. My left arm is heavy and warm. My left arm is heavy and warm.

Observe the sensations in the left arm for a moment. I feel calm and relaxed. Now include both hands and arms in your awareness and repeat silently: My arms are warm and heavy. My arms are warm and heavy. My arms are warm and heavy. My arms are warm and heavy. My arms are warm and heavy.

Feel your arms generating a warm, relaxing wave that spreads through your entire body. Now focus on your right leg and repeat: I feel calm and relaxed. My right leg is heavy and warm. My right

`

leg is heavy and warm. My right leg is heavy and warm. My right leg is heavy and warm. My right leg is heavy and warm.

Attend for a few more seconds to the sensations in your right leg, then gently and slowly shift your focus to your left leg, repeating after me: I feel calm and relaxed. My left leg is heavy and warm. My left leg is heavy and warm. My left leg is heavy and warm. My left leg is heavy and warm. My left leg is heavy and warm.

Include both legs in your awareness and repeat: I feel calm and relaxed. Both of my legs are heavy and warm. Both of my legs are heavy and warm. Both of my legs are heavy and warm. Both of my legs are heavy and warm. Both of my legs are heavy and warm.

Bring your attention to your arms and legs together and repeat silently: I feel calm and relaxed. Notice the sense of warmth and heaviness throughout the body. Observe whether these sensations change in intensity—perhaps one area feels especially warm, another heavier than the rest. Observe your body without trying to change anything you feel. I feel calm and relaxed. Sense your body becoming completely relaxed and gently drowsy.

Listen to your heart and repeat: I feel calm and at ease. Repeat silently: My heart beats calmly and evenly. My heart beats calmly and evenly. My heart beats calmly and evenly. My heart beats calmly and evenly. My heart beats calmly and evenly. Now move your attention slightly upward to your head and feel a pleasant coolness spreading across your forehead. Repeat in your mind: My forehead is cool and I feel relaxed. My forehead is cool and I feel relaxed. My forehead is cool and I feel relaxed. My forehead is cool and I feel relaxed. My forehead is cool and I feel relaxed.

Notice how different sensations can give you a profound sense of calm and relaxation. Now scan what you feel in your body, from head to toe. Repeat silently after me: My forehead is cool and I feel relaxed. My forehead is cool and I feel relaxed. My heart beats calmly and evenly. My heart beats calmly and evenly. My abdomen is pleasantly warm and relaxed. My abdomen is pleasantly warm and relaxed. My arms are heavy and warm. My arms are heavy and warm. My legs are heavy and warm. My legs are heavy and warm.

Immerse yourself in all of these pleasant sensations.

We have now reached the end of today's practice. I hope this recording has helped you unwind and find calm. Please remember: relaxation techniques and meditation are practices—repetition makes us better at them, and with that we gain greater control over our bodies and minds. If you do not feel a clear change today, give yourself time. Do not rush anything—at some point you may be surprised by how much your sensations can shift. Thank you, and see you next time.

Technical specification:

Collection of relaxation videos with voiceover sound, therapeutic music, nature sounds, each video lasts ~10 minutes.

Display: 5.46" LCD screen.

Resolution: 3664x1920, 4K standard.

Refresh rate: 75Hz/90Hz

Manual IPD adjustment: 56/63.5/69 mm

Field of view: 98°

Battery life: ~2,5 h

Battery charging time: ~110 min

Port: USB-C

Dimensions: 86mm x 166mm x 90mm

Color: white/gray

Weight: ~604g

`

Table S1. Parameters of the VR session.

Title	Session type	Nature sounds	Therapeutic narration	Therapeutic music	Duration
State of Balance	Breathing session, 4-4-4-4	Yes	Yes	Yes	10:36
The art of Mastery	Breathing session, 4-7-8	Yes	Yes	Yes	10:39
Mountain valley	Mindfulness breathwork session	Yes	Yes	No	10:06
Forest hike	Mindfulness breathwork session	Yes	Yes	No	10:25
Seaside beach	Mindfulness breathwork session	Yes	Yes	No	10:25
Snowy lane	Mindfulness breathwork session	Yes	Yes	No	12:05
Therapeutic rebirth	Relaxation meditation session	Yes	Yes	Yes	11:25
Rocky coast	Mindfulness breathwork session	Yes	Yes	Yes	9:59
Underwater world	Mindfulness breathwork session	Yes	Yes	No	10:35
Serenity of the Lake	Mindfulness breathwork session	Yes	Yes	No	10:18
Dawn meditation	Relaxation meditation session	Yes	Yes	Yes	11:21
Twilight meditation	Relaxation meditation session	Yes	Yes	Yes	15:24
Breathing training	Mindfulness breathwork session	Yes	Yes	Yes	14:27
Walk in the clouds	Mindfulness breathwork session	Yes	Yes	Yes	10:34
Natural soothing	Breathing session, 4-2-8	Yes	Yes	Yes	10:06

Session description

State of Balance - Stop by the river for a moment to notice how life flows within you with each wave of breath. The freshness of the landscape will improve your vitality, and the harmonious environment of nature will allow you to breathe more calmly. Consciously balancing your breathing will invite harmony into your interior and reduce anxiety. If you want to balance stress and achieve an inner State of Balance I invite you to this breathing session.

The art of Mastery - Breathing enables you to change your emotional state, and is crucial to your well-being. In this delightful place where nothing distracts you, pause over The Art of Mastery. A carefully executed breathing cycle will relieve your body of stress and put you in a state of pleasant relaxation. With each carefully crafted breath, you will strengthen the foundation of life and your connection to nature.

Mountain valley - Once the snow from the nearby peaks has melted and fed the mountain river, it is a good time to safely admire the majesty of the mountains from the picturesque valley. Breathe, let fresh air bring you lightness and new energy. Entrust your worries to the silent mountains and let your fears flow away through with the swift river. Find hope among the juicy greenery. Soak up the beauty of the Mountain Valley with your whole self. Let your life be like this.

Forest hike - Relax in your trek right here, surrounded by young birches and tall pine trees. Let the invigorating gust of wind will move your inside and blow away your dark thoughts. Take a deep breath in this forest bath, feel the forest with all of you. Discover that nature took its time to create this beautiful forest during your Forest hike. You can also create a beautiful life, by taking your time.

Seaside beach - The beach is an unusual place where some things end and some begin. Looking with hope at the horizon, let the waves of the sea wash away the traces of sadness and stress that you carry inside you. Relax. Keep the harmonious rhythm of the waves and the warm rays of the sun that bring blissful peace in your mind. Whenever you feel you are in a worse shape, return to the Seaside Beach to relax and restore your well-being

Snowy lane - You came here first, so this undisturbed snowy view is all yours. Relax your eyes in complete silence, looking at the sheer beauty of the snowy trees. Keep the stunning riches of this moment in your memory. Before returning to the old tracks of noise and haste, breathe and regain your energy for action. Remember that if you are in need, the Snowy Lane is waiting for you to follow it back into the enveloping winter landscape.

`

Therapeutic rebirth - Behind the garden gate, you can relax and feel a surge of strength. Take a slow look around and listen to the sounds of the scenic garden space. You can grow and flourish together with the surrounding plants. Breathe and let the surrounding beauty penetrate into you, bringing you balance and well-being. Open your senses to the good. Let Therapeutic Rebirth be part of your experience.

Rocky coast - The sea waves carry energy, and the brisk breeze refreshes the view of many things. Even small clouds can herald changes, but when you have a firm foundation under your feet, you don't have to worry about anything. Standing on the stable Rocky Coast, open yourself up to a harmonious surge of power. Let the water wash away your worries, experience the lightness. Look afresh into the distance and breathe in good weather. It's all fine now, and let's keep it that way.

Underwater world - Change your perspective and immerse yourself in the depths of the sea. Leave your problems on the shore and sail away into this immersive experience. In underwater silence, you can easily focus on your own breath and indulge in relaxation. Admire the Underwater World, find your shelter here. A delightful visual experience will bring you peace and tranquillity, which will stay with you for a longer time. If you like this place, drop your anchors right here.

Serenity of the Lake - The sun rose early today to share its energy with nature. Pleasant warmth invites you to contact with nature. Pause at the calm surface of the lake and observe the soothing landscape. You'll feel safe under the sprawling tree. Breathe consciously. Even a gentle movement of the air can move the water, just as your breath can move your body. Give yourself a moment of rest. Just be here and feel the Serenity of the lake.

Dawn meditation - If you want to feel calm and in control of your body and mind, try a proven way to total peace. It may be difficult for you to find the right time and place for this. But here is a perfect space, where nothing distracts you in the search for harmony. Sit comfortably in the middle of a beautiful garden and concentrate on your own breath. Start with the short Dawn Meditation. For a good day and a good life without stress.

Twilight meditation - Get rid of tension in a wonderfully peaceful garden that exudes good energy and invites you to calm down. Try the Twilight Meditation to get rid of the anxiety and stress that is blocking you. You will gain inner peace, improve concentration and your ability to make decisions. Truly notice your breath, and once you are able to control it, peace, balance, health and happiness will appear in your life. All the best things.

Breathing training - Your breath lets you live. It happens on its own, so you don't think much about it. During the Breath Training, you can notice your breath again. You will learn to use your breath consciously for your health and well-being. In the Virtual Garden of Rebirth, you can train the way you breathe so that it becomes perfect, giving you energy and strength for further action. Give yourself a break, it's good for you.

Walk in the clouds - Deep breathing is the source of good for your body and mind. Go for an oxygenating Walk in the clouds that will bring you well-being and serenity. In this scenery, you will feel one with nature, which will enable you to restore balance. Your conscious breath will sweep away anxiety, and everything that puts you down, like the wind sweeps away the clouds. You can see the passing of clouds every day, you just have to go for a walk.

Natural soothing - Breath is a manifestation of life and your condition, it can give you energy and bring Natural Soothing. You have it with you at all times. Together with the VR TierOne GO in a conducive environment, you can learn to make full use of your breath for your health. The pleasant experience of Virtual Reality and the practice of conscious breathing will allow you to relax and feel better in your tension-free bod.

The session descriptions come directly from VR TierOne's official website - https://vrtierone.com/products/vr-tierone-go-mobile-relaxation-headset/

Table S2. Descriptive statistics for the whole study sample.

`

Variable	Valid n	Mean	Minimum	Maximum	Std. deviation	Skewness	Kurtosis
HADS – anxiety, before	37	12.892	3	21	4.795	-0.668	-0.285
HADS – anxiety, after	37	10.054	1	18	4.301	-0.274	-0.047
HADS depression, before	37	10.135	0	19	4.995	-0.311	-0.642
HADS depression, after	37	8.216	0	19	5.039	0.248	-0.803
AIS, before	37	10.027	2	22	4.752	0.588	-0.188
AIS, bfter	37	6.405	0	15	3.670	0.281	-0.389
MOCA, before	37	25.838	13	30	4.781	-1.385	0.938
MOCA, after	37	26.865	16	30	3.713	-1.557	2.198
ACE-III – attention, before	37	16.946	12	18	1.682	-1.687	1.916
ACE-III – memory, before	37	21.919	8	26	5.669	-1.383	0.501
ACE-III – fluency, before	37	12.135	5	14	2.626	-1.721	1.842
ACE-III – language, before	37	25.108	16	26	2.196	-2.975	9.002
ACE-III – visualspatial functions, before	37	14.297	9	16	1.956	-1.314	1.410
ACE-III – total score, before	37	90.405	61	100	11.784	-1.525	0.921
ACE-III – attention, after	37	17.243	12	18	1.623	-2.149	3.468
ACE-III – memory, after	37	23.568	10	26	3.476	-2.017	5.251
ACE-III – fluency, after	37	12.405	5	14	2.315	-1.719	2.339
ACE-III – language, after	37	25.622	22	26	1.063	-2.828	6.977
ACE-III – visuospatial functions, after	37	14.351	8	16	2.031	-1.435	1.724
ACE-III – total score, after	37	93.189	67	100	8.306	-1.580	2.001

`

Figure S1. HADS anxiety scores before and after the interventions in the whole study sample (N = 37).

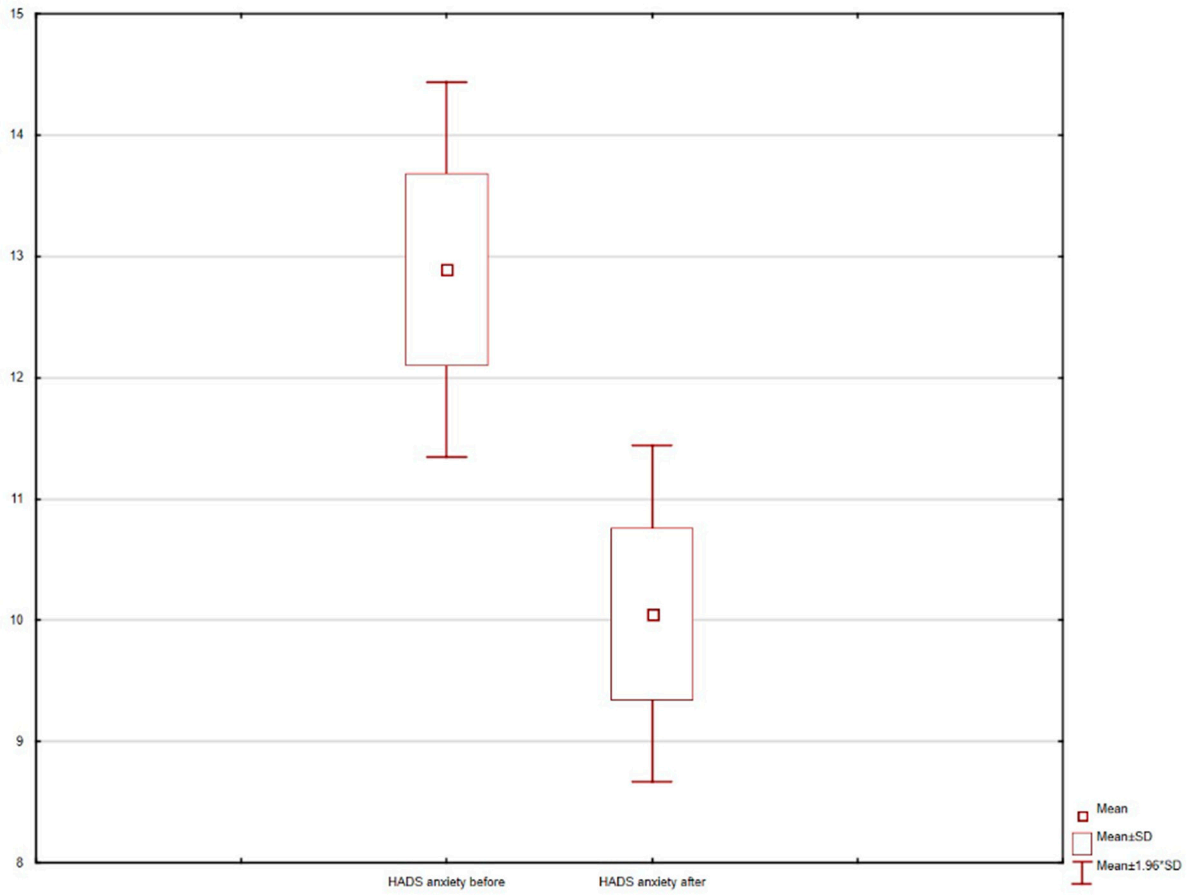

`

Figure S2. HADS depression scores before and after the interventions in the whole study sample (N = 37).

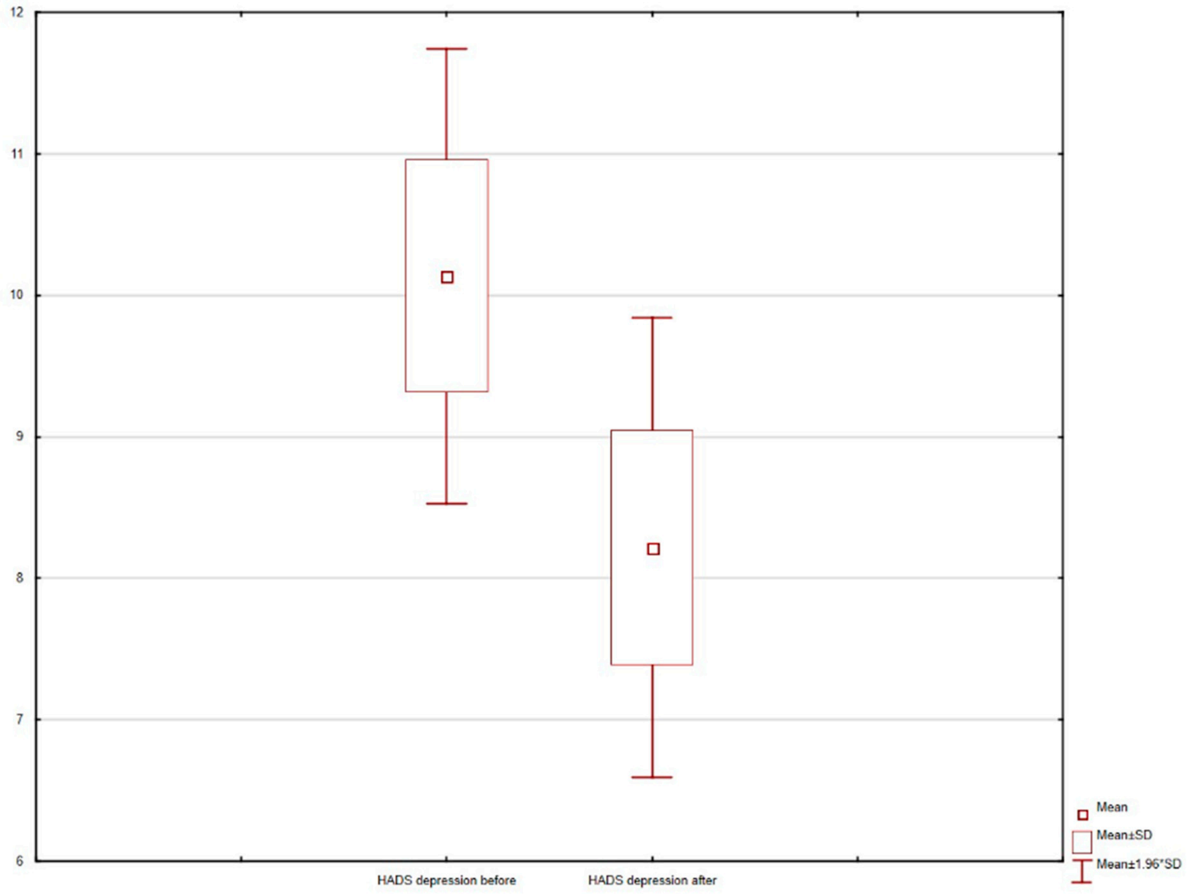

`

Figure S3. AIS scores before and after the interventions in the whole study sample (N = 37).

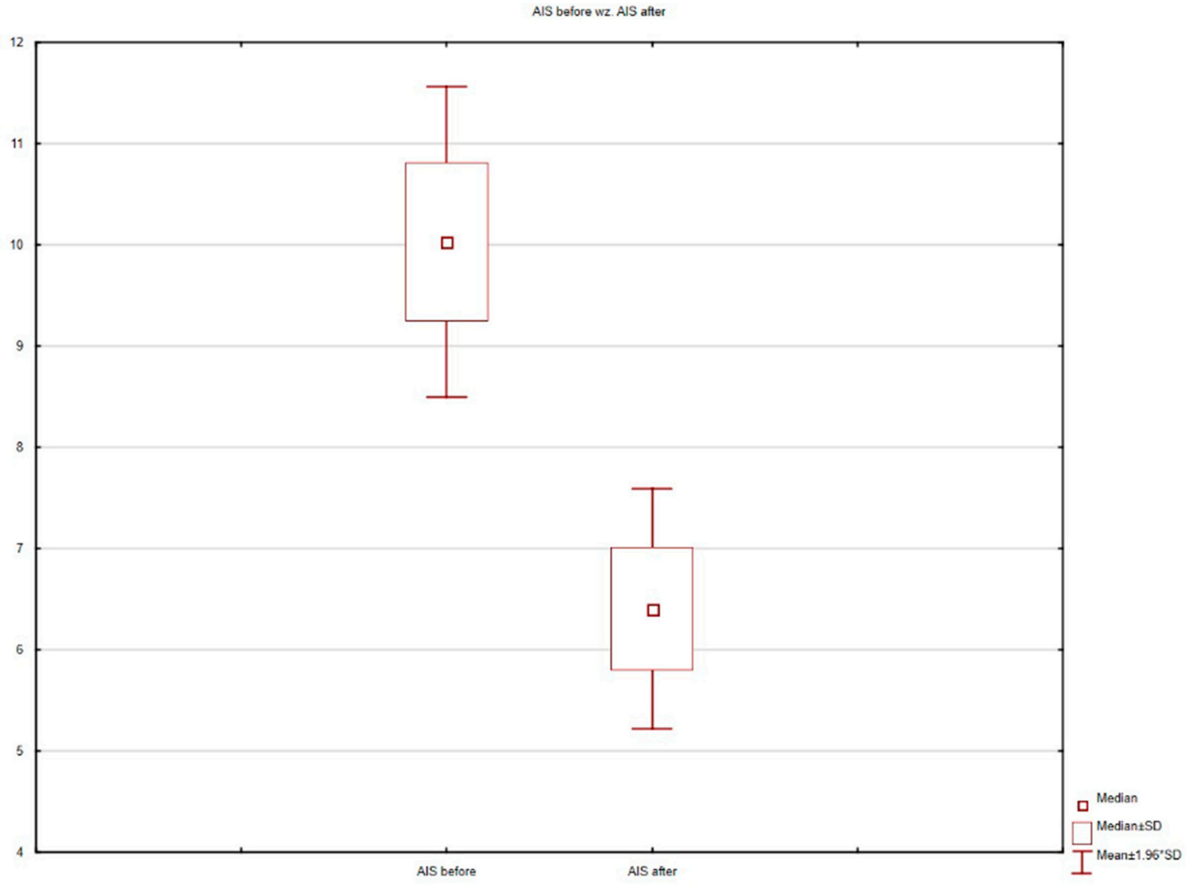

`

Figure S4. MOCA scores before and after the interventions in the whole study sample (N = 37).

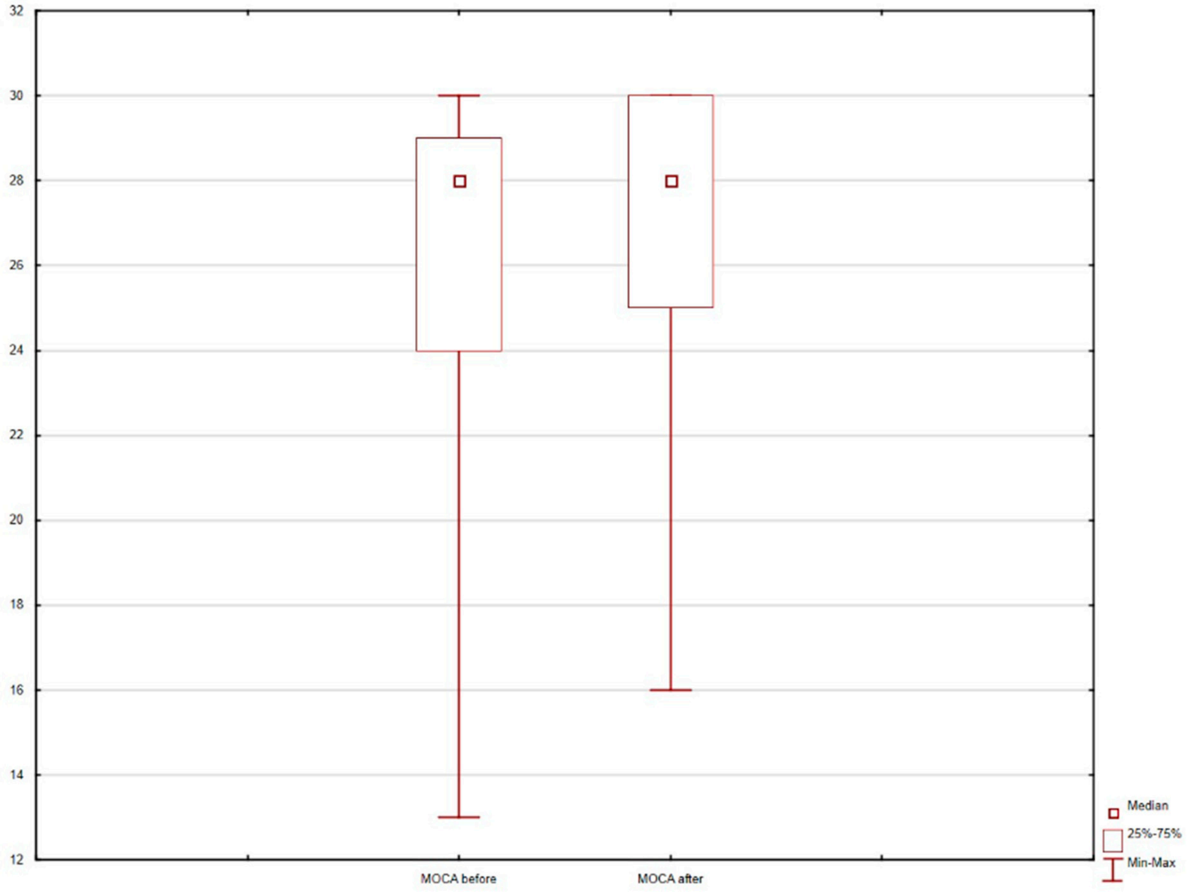

`

Figure S5. ACE-III scores before and after the interventions in the whole study sample (N = 37).

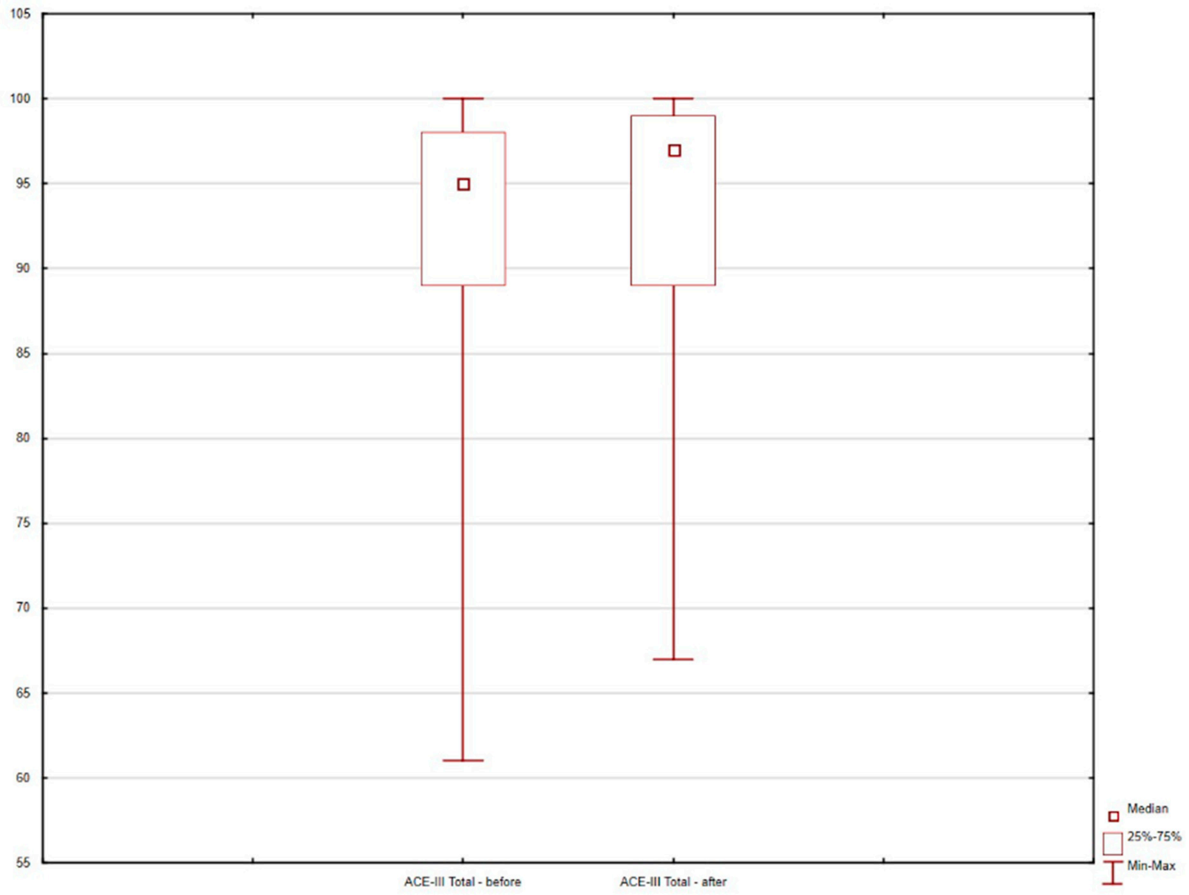

Supplement: Supplementary file 1 [file reports-08-00247-s001.zip › reports-3968758-supplementary.pdf]
